# The Interplay of Quantum Confinement and Hydrogenation in Amorphous Silicon Quantum Dots

**DOI:** 10.1002/adma.201503013

**Published:** 2015-11-02

**Authors:** Sadegh Askari, Vladmir Svrcek, Paul Maguire, Davide Mariotti

**Affiliations:** ^1^ Nanotechnology & Integrated Bio‐Engineering Centre‐NIBECUlster UniversityNewtownabbeyBT37 0QBUK; ^2^ Research Center for Photovoltaic TechnologiesNational Institute of Advanced Industrial Science and Technology‐AISTCentral 2Umezono 1‐1‐1Tsukuba305‐8568Japan

**Keywords:** amorphous silicon, hydrogen alloying, photoluminescence, quantum dots

## Abstract

**Hydrogenation in amorphous silicon quantum dots (QDs)** has a dramatic impact on the corresponding optical properties and band energy structure, leading to a quantum‐confined composite material with unique characteristics. The synthesis of a‐Si:H QDs is demonstrated with an atmospheric‐pressure plasma process, which allows for accurate control of a highly chemically reactive non‐equilibrium environment with temperatures well below the crystallization temperature of Si QDs.

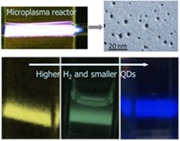

Silicon (Si) nanostructures are attracting considerable attention for a wide range of applications^1^, ^2^, ^3^, ^4^, ^5^, ^6^ due to their fascinating and highly complex properties.^7^, ^8^, ^9^ For instance, efficient photoluminescence (PL) from crystalline Si quantum dots (i.e., with diameter less than ≈5 nm) has been reported, which encourages their applications in optoelectronic devices such as light‐emitting diodes^10^ or in biomedical applications as fluorescent tags.^11^ Further properties originating from the strong quantum confinement regime such as carrier multiplication have been also reported,^12^, ^13^ which makes them very attractive for integrating Si quantum dots (QDs) in photovoltaic cells.^1^

While crystalline Si (c‐Si) QDs have been extensively studied within the last decade,^14^, ^15^, ^16^, ^17^, ^18^, ^19^ investigation of amorphous Si (a‐Si) QDs properties has been very limited. One of the reasons for this limited effort is represented by the synthesis difficulties: during synthesis, it is challenging to preserve both the individuality of the QDs and their amorphous nature as QDs tend to aggregate with consequent loss of the quantum‐confined state. Nonetheless, the quantum‐confined state of a‐Si is of great scientific interest as demonstrated by the vast literature that addresses advantageous and complementary differences between bulk a‐Si and bulk c‐Si.^20^ For instance, in the bulk form, higher PL efficiency has been reported for a‐Si compared to c‐Si. This is due to the enhanced structural disorder in a‐Si.^21^ Furthermore, it is well known that alloying a‐Si with hydrogen (a‐Si:H) in the bulk form can passivate the dangling bonds and dramatically improve the optical and electronic properties; hydrogen inclusion can reduce defect density by orders of magnitude^22^ and it can widen the bandgap.^22^, ^23^, ^24^ The hydrogen content has been shown to be a reliable control parameter for tailoring the bandgap,^22^, ^23^, ^24^ allowing wider bandgap energies for a‐Si:H compared to c‐Si (1.1 eV), which is very attractive for tunable optical properties at shorter wavelengths. Therefore, similar opportunities may be available for a‐Si and a‐Si:H QDs; in combination with other nanoscale properties (e.g., quantum confinement, surface‐to‐volume ratio, surface chemistries etc.), these QDs can provide added functionalities not available from bulk a‐Si/a‐Si:H and also complementary to c‐Si QDs. For instance, a‐Si:H QDs can represent a mean for biodegradable drug delivery with a photoluminescent signal that is intrinsically linked to the degradation state.^25^, ^26^

The study of a‐Si nanoparticles (NPs) has been often limited to particles embedded within a bulk matrix (e.g., silicon nitrides).^27^ NPs within bulk materials present very different characteristics compared to “free‐standing” NPs or QDs: these include fundamental differences (e.g., matrix‐induced mechanical strain, phonon interactions), different synthesis challenges (e.g., hydrogenation is challenging to control within a bulk matrix) and different application focus (e.g., not suitable for applications that require colloids). Free‐standing a‐Si NPs have been synthesized using vacuum/low‐pressure plasmas^28^, ^29^, ^30^ to verify, for instance, the intermediate stage leading to the formation of c‐Si NPs and the effect of quantum confinement and hydrogenation has not been so far addressed. Furthermore and importantly, in all the above studies the investigations have been limited to NPs with diameters greater than ≈4 nm, i.e., NPs that are too large to exhibit properties originating from the strong quantum confinement regime.

Here, we report the experimental study on the synthesis of free‐standing a‐Si QDs (i.e., within the strong quantum confinement regime), their optical properties and impact on the corresponding energy structure. Accurate control over the size of QDs was achieved using a unique atmospheric‐pressure microplasma reactor, which allowed investigating the effect of QDs size on the PL. Furthermore and importantly, this new synthesis methodology enabled the synthesis of a‐Si QDs for the first time with varying hydrogen content (i.e., a‐Si:H QDs), allowing the determination of the effect of hydrogen on the optical properties. The synthesis of QDs by atmospheric‐pressure plasma has allowed effective hydrogen incorporation and ease of sample handling with limited oxidation.

A schematic diagram of the plasma reactor is depicted in **Figure** **1**. **Figure** **2**a shows a representative transmission electron microscopy (TEM) image of the QDs produced with this reactor. The reactor is designed for operating at atmospheric pressure and it is applicable to the synthesis of a range of QDs including c‐Si QDs.^19^ With this reactor design, steady‐state cold plasmas with variable gas temperature down to room‐temperature can be generated and controlled. Although the gas temperature is low and constant, the population density of energetic electrons is high, which results in a highly reactive chemical environment capable of efficient precursor dissociation and QDs formation. One of the advantages of this approach is that by simply controlling a range of parameters we can for instance select between amorphous and crystalline structure of the synthesized QDs. For the synthesis of amorphous QDs and effective hydrogenation, the gas temperature needs to be kept relatively low as this affects directly the temperature of the growing NPs;^19^ a higher gas temperature, and therefore even higher NP temperatures, would contribute to both hydrogen desorption during nucleation and growth as well as crystallization. As depicted in Figure 1, the QDs form in‐flight in the direction of the gas flow, therefore the growth can be adjusted by varying the time that the QDs spend within the plasma, i.e., the residence time. The low residence time (< ms) achievable in this reactor allows controlling and accurately limiting the size of the QDs down to less than 2 nm in diameter. The impact of the reactor configuration on the plasma properties is discussed in the Supporting Information.

**Figure 1:**
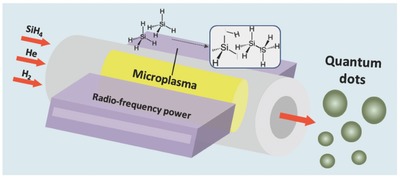
Schematic diagram of the microplasma reactor employed for synthesis of amorphous Si quantum dots.

**Figure 2:**
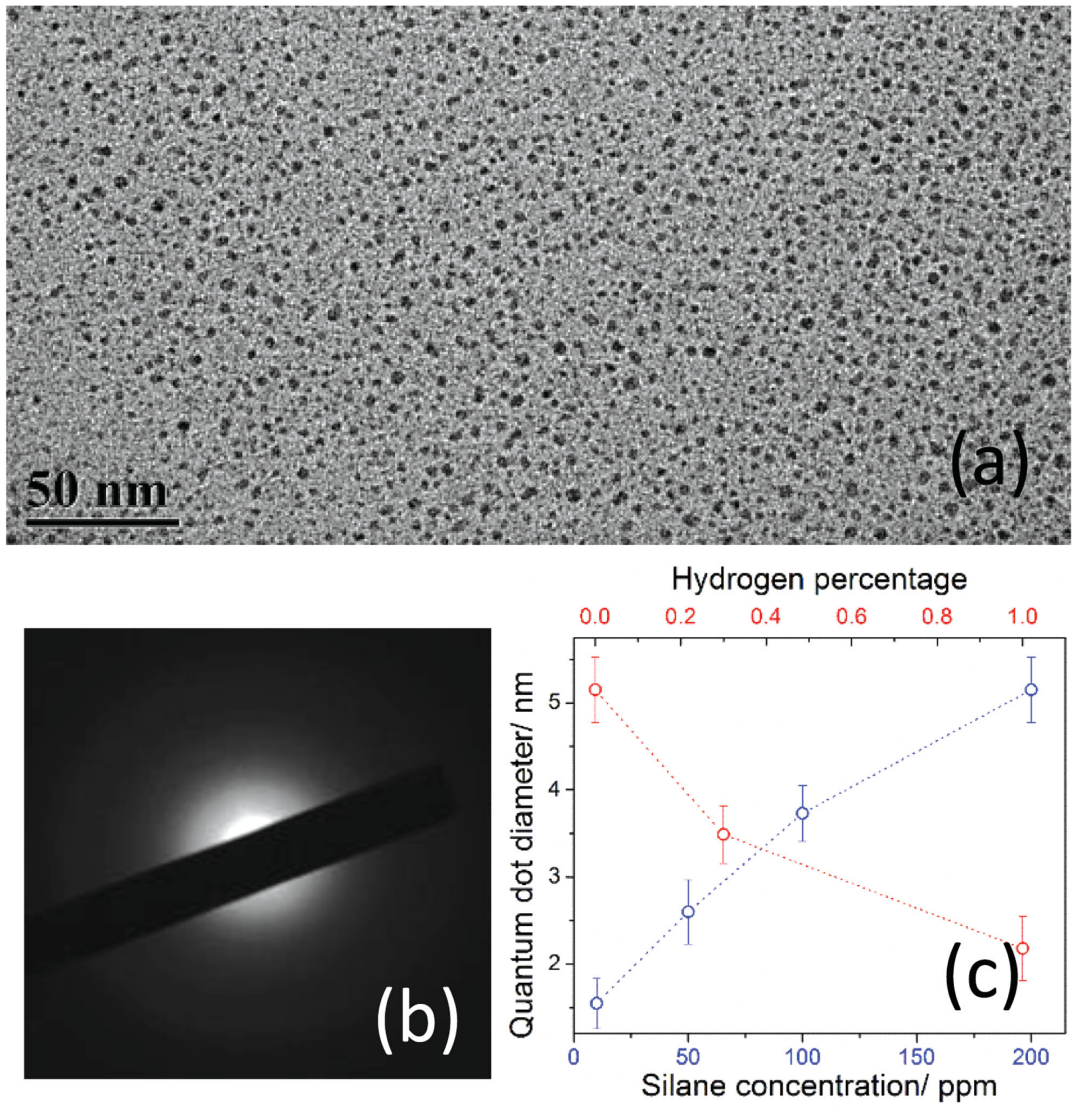
a) Transmission electron microscopy (TEM) image of a‐Si quantum dots (QDs) produced with 50 ppm silane. b) The corresponding diffraction pattern confirms the amorphous structure of the QDs. c) Mean value of the QDs diameter prepared at several different silane concentrations (with no hydrogen added) and hydrogen dilutions (with 200 ppm constant silane); the error bars represent the standard deviation of the distributions for the corresponding mean values.

In the reactor, the plasma is sustained by applying 100 W radio frequency (RF) power at 13.56 MHz to the powered electrode through an automatic matching unit (ENI MWH‐5‐01m9). Electrodes, made of brass, are positioned at both sides of a quartz capillary with an internal diameter of 0.7 mm and an external diameter of 1 mm. The electrodes are 10 mm long in the quartz tubing axial direction and 1 mm thick covering the full diameter of the capillary (Figure 1). The plasma reactor is housed in a nitrogen‐filled sealed stainless‐steel chamber in which the pressure is maintained at one atmosphere. A gas mixture of helium (He), argon (Ar), silane (SiH_4_), and hydrogen (H_2_) is used here and flows through the capillary. Silane is supplied from a gas bottle that contains 0.54% silane diluted in Ar while the hydrogen bottle is 1.0% in helium background. The total flow of the gas into the reactor is fixed at 800 sccm while the silane and H_2_ concentrations are varied to produce different synthesis conditions: 10, 50, 100, and 200 ppm for silane and 0%, 0.3%, and 1.0% for H_2_.

Thanks to the atmospheric‐pressure operation, the QDs produced in the plasma can be collected downstream of the reactor directly in colloids or as powder or directly deposited on substrates for direct integration in application devices. Within this specific study, we have collected the QDs in a vial containing ethanol or deposited directly on stainless‐steel substrates. TEM analysis of the QDs has been performed with a JEOL JEM‐2100F; a few drops of the QDs/ethanol colloids are drop‐cast on TEM grids in order to analyze each sample. Mean diameter of the QDs and size distributions were determined throughout this work by TEM analysis (see corresponding size distribution histograms in the Supporting Information). The chemical analysis of the samples has been performed by Fourier transform infrared (FTIR) spectroscopy using an attenuated total reflectance (ATR) accessory. The ATR–FTIR spectrometer is a Nicolet iS5 from Thermo Scientific, and for the corresponding measurements, the collected sample powder on a stainless‐steel substrate is placed on the top of the ATR crystal. PL measurements were carried out with an Ocean Optics QE65 Pro and the excitation source was an LED with wavelength at 365 nm (Ocean Optics LLS‐LED). UV‐vis absorption measurements are performed using a Perkin–Elmer, Lambda 35 UV–VIS. Samples for PL and UV‐vis measurements are prepared by collecting the QDs in high purity ethanol (impurity < 1 ppm; Sigma–Aldrich). Ethanol was degassed to remove oxygen content (with helium) for ≈10 min before being used. Synthesis within an inert environment and collection directly in ethanol has allowed us to minimize oxidation of our QDs, which are expected to be H‐terminated. It is important to note that Si–H bonds are very stable in dry air up to 500 °C and oxidation is mainly due to water or water vapor.^31^ Therefore the oxidation of Si NPs and QDs in ethanol is slowed down compared to exposure to humid air (or storage in water); for instance for crystalline silicon oxidation becomes significant only with long‐term (hours to days) exposure to water or humid air.^32^ All measurements presented here were carried out shortly after synthesis (<1 h) therefore preserving the as‐synthesized surface characteristics (see further below for the material characterization). This will be further confirmed with our surface analysis below, which shows a very low degree of oxidation.

Figure 2a displays a typical TEM image of QDs produced with 50 ppm silane. We have been unable to find any crystalline structure in all of the samples that we have analyzed by TEM and the diffraction pattern (Figure 2b) consistently confirms that the QDs are amorphous. This is further corroborated by Raman spectroscopy (see Supporting Information). We have therefore produced QDs with 10, 50, 100, and 200 ppm silane. The detailed material characterization has demonstrated that a‐Si QDs are produced at all four different synthesis conditions (see Figure 2a,b and Supporting Information); due to silane, which is used as precursor, H‐terminated QDs are produced with a degree of hydrogenation within the QD cores. Statistical analysis using TEM shows that by fitting the experimental results with a log‐normal distribution, the mean diameter was found to be 1.55, 2.60, 3.73, and 5.15 nm, respectively (circles in Figure 2c; see also Supporting Information). The relationship between NP size and precursor concentration has been long established for synthesis techniques in low‐pressure plasmas.^33^

We now focus on the optical properties of the a‐Si QDs. In order to demonstrate the effect of quantum confinement on the optical bandgap, UV‐vis absorption measurements were carried out for the colloids of QDs with silane concentration 10–200 ppm (see Supporting Information). The absorption gap is estimated assuming indirect bandgap (see also Supporting Information).^22^, ^23^, ^24^ This yields values of 3.22, 3.11, 2.91, and 2.58 eV for the bandgaps of corresponding QDs with mean diameter of 1.55 nm (10 ppm), 2.60 nm (50 ppm), 3.73 nm (100 ppm), and 5.15 nm (200 ppm), respectively, which confirm the bandgap widening due to quantum confinement.

**Figure** **3**a shows the normalized PL profiles of the samples with different concentration of silane. The emission was stable for all samples over time (>10 min) confirming that oxidation in ethanol was relatively slow. The PL peak wavelength is red‐shifted with increasing silane concentration: 463, 517, 580, and 605 nm for 10, 50, 100, and 200 ppm silane, respectively. The corresponding mean diameter of QDs (obtained from TEM) is also shown with the PL profiles in Figure 3a. The PL peak is red‐shifted by about 0.7 eV with increasing the QD diameter in the range 1.55–5.15 nm, which is consistent with quantum confinement as already supported by the absorption measurements (see Supporting Information). The difference between the optical bandgap from absorption measurements and the PL peak wavelength, known as Stokes shift, is relatively small (0.55–0.77 eV) as large values of the Stokes' shift (>1.3 eV) have been reported for crystalline Si NPs.^34^, ^35^

**Figure 3:**
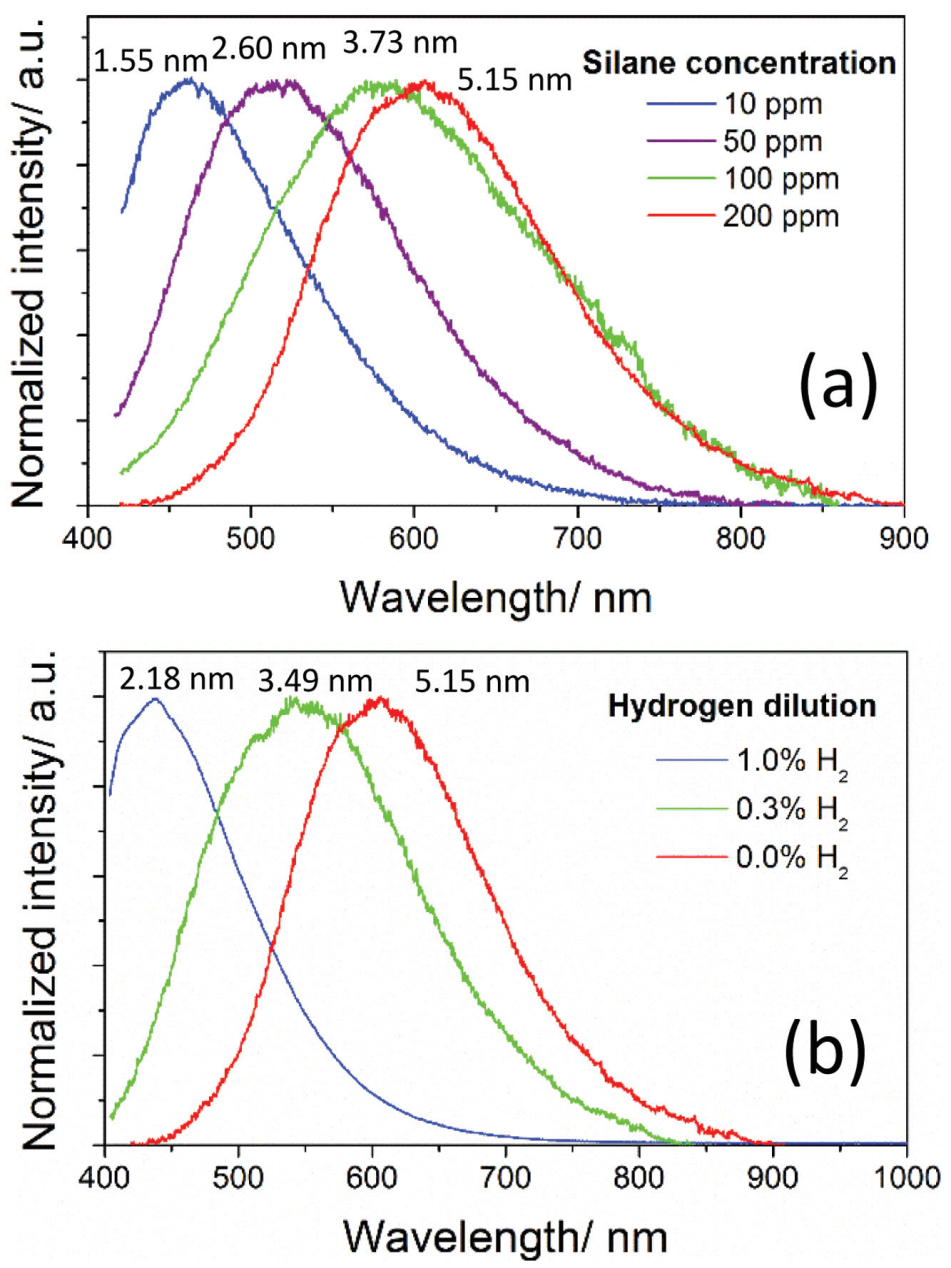
a) Normalized photoluminescence spectra at four different concentrations of silane in the range from 10 to 200 ppm. The average size of the quantum dots is increased from 1.55 to 5.15 nm with increasing silane concentration. b) Normalized photoluminescence spectra for quantum dots with three different concentrations of additional H_2_ in the plasma (200 ppm silane). The mean diameter of the quantum dots is decreased from 5.15 to 2.18 nm with increasing H_2_ concentration.

The synthesis of a‐Si QDs was then performed also with added hydrogen at varying concentrations (0%, 0.3%, and 1.0%) to 200 ppm silane. The QDs remained amorphous (see detailed material characterization in the Supporting Information) and their mean diameter is reduced from 5.15 nm (no hydrogen) to 3.49 nm for 0.3% added hydrogen and 2.18 nm for 1.0% added hydrogen (Figure 2c). The reduction in the size of the QDs with increasing hydrogen can be attributed to the etching of particles in the plasma by hydrogen radicals; adding hydrogen to a plasma is a well‐known method for dry etching of Si‐based materials.^36^ In part, silane dissociation efficiency is also reduced with added hydrogen due to a fraction of the electron energy being diverted into hydrogen dissociation; this could have also contributed to a size reduction as observed in Figure 2c.

FTIR analysis of a‐Si QDs samples was carried out to better understand the impact of added hydrogen in the chemical composition of the QDs. The full FTIR spectra of the samples prepared with 200 ppm silane and different H_2_ percentages (0.0%, 0.3%, and 1.0%) are reported in the Supporting Information; **Figure** **4** presents the analysis of the stretching band at 1950–2225 cm^−1^. This band is attributed to silicon hydrides bonds (Si–H*_x_*, *x* = 1, 2, 3) where the position of the peak is at higher wavenumbers for higher order hydrides.^37^, ^38^ The deconvolution of the profiles in Figure 4 is performed by fitting Gaussian curves corresponding to Si–H (2070 cm^−1^), Si–H_2_ (2105 cm^−1^), and Si–H_3_ (2145 cm^−1^) following reports for hydrogenated silicon.^37^, ^38^ It is clear that the contribution of higher order hydrides increases with increasing added hydrogen concentration, indicating the unambiguous impact in the coordination degree of Si–H bonds. The hydrogen can be present within the QD and/or on its surface. While for bulk a‐Si:H, the impact of hydrogen‐bonded surfaces is minimal, for a‐Si QDs, the absorption from H‐terminated surface can be considerable and therefore it is not straightforward to discern surface H‐terminations from hydrogen incorporation in the QDs core. The FTIR results, however, confirm that increasing hydrogen concentration in the plasma process is effective in increasing the hydrogen content in and on the QDs. Hydrogen incorporation is also indicated by the increasing overall absorption of the H‐related peaks with increasing added hydrogen, while the overall absorption intensity of the Si–H*_x_* peaks did not vary significantly for the Si QDs in the diameter range 1.55–5.15 nm when hydrogen gas was not used in the synthesis process (see Supporting Information).

**Figure 4:**
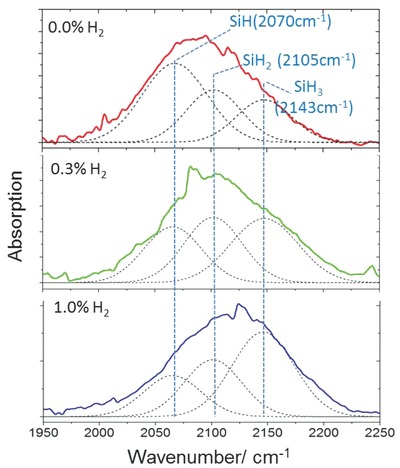
The stretching band silicon hydrides at 1950–2250 cm^−1^ of the infrared spectra of the amorphous Si quantum dots prepared at silane concentration 200 ppm and three different H_2_ dilution 0.0%, 0.3%, and 1.0%. The ratio of the higher hydride increases at higher concentration of H_2_.

PL measurements were also carried out for the samples prepared with additional hydrogen. Figure 3b displays the PL profiles measured for these samples with three different percentages of added hydrogen, 0%, 0.3%, and 1% and with 200 ppm silane. The position of the PL peak is blue shifted from 605 nm, to 548 nm and to 438 nm with increasing H_2_ concentration, which correspond to a‐Si QDs with different mean diameter (see Figure 3b). The PL measurements confirm quantum confinement effects also in these QDs with added hydrogen.

The interplay of quantum confinement and hydrogenation in the bandgap of the QDs can be studied with the help of theoretical models. Different theoretical approaches have been employed including effective mass approximation (EMA), tight binding approximation, and empirical pseudopotential plane wave theory that do not always lead to the same results. According to the widely used EMA, the increase in the bandgap *∆E* (*∆E = E − E*_0_, where *E* and *E*_0_ are the bandgap energy of QD and bulk, respectively) is proportional to the inverse square of the QD diameter, *∆E = A/d^δ^*, with a proportionality coefficient *A* and *δ* = 2. Within the EMA, the confinement factor *A* = 13.5 has been reported for c‐Si QDs.^39^ The applicability of EMA to amorphous systems and in particular to a‐Si NPs has been analyzed and discussed by Barbagiovanni et al.^40^ revealing that “strong confinement” can describe amorphous materials. Therefore, in the case of a‐Si QDs with diameter down to ≈2 nm^41^ and within the strong confinement due to the spatial delocalization of holes, values of A in the range 1.39–3.57 have been suggested.^40^

In **Figure** **5**, the PL peak energy representing the QDs bandgap (see Supporting Information) versus mean QD diameter is shown along with the expected fit from EMA. Without added hydrogen (see blue squares and blue full curve in Figure 5), an initial bulk bandgap of *E*_0_ = 1.97 eV and confinement factor *A* = 2.14 have been obtained; these are values that are close to those reported from experimental measurements for amorphous hydrogenated silicon and a‐Si QDs.^22^, ^23^, ^24^, ^40^ Our data point for 1.55 nm diameter QDs is in principle just below the limit of the EMA applicability and it may be the cause for the slight divergence at lower bandgap values. Even without added hydrogen, a degree of hydrogenation is to be expected where the hydrogen concentration, originating from silane, is predicted to be constant for different QDs sizes. The theoretical curve for the QDs with added H_2_ (red full curve in Figure 5) is produced by assuming a higher and increasing bulk bandgap value (*E*_0_) to reflect the increasing hydrogenation; it is well studied and accepted that adding hydrogen effectively changes the bulk optical bandgap, which is expected to be even higher than 2.0 eV in bulk a‐Si:H.^22^, ^23^ In fact, the higher curvature presented by the round red symbols in Figure 5 could be interpreted only with an increasing value for *E*_0_, which corresponded to 2.09 eV and 2.38 eV for a‐Si QDs with diameter of 3.49 nm and 2.18 nm respectively (see Supporting Information); the increasing bulk bandgap used in the EMA fitting describes adequately the higher degree of hydrogenation (see red full line in Figure 5). This can support the hypothesis that a larger bandgap for the a‐Si QDs with added hydrogen may be due to hydrogen incorporation in the QD cores rather than just at the surface.

**Figure 5:**
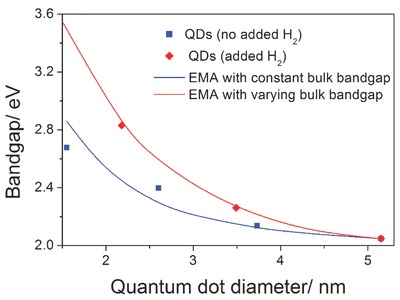
Bandgap of hydrogenated amorphous silicon quantum dots versus their diameter; blue and red squares represent the quantum dots prepared at different concentrations of silane and H_2_, respectively. The solid curves plot the band energy calculated from effective mass approximation (EMA) (see Supporting Information for full details on the fitting procedure).

A method for the synthesis of a‐Si QDs with control over size and hydrogen content was reported. QDs within the strong confinement regime with average diameters in the range 1–6 nm were synthesized where the control over size was possible through changing either silane or hydrogen concentration. The demonstrated synthesis method can be employed for the synthesis of other materials QDs. The PL of a‐Si QDs was studied and its dependence on size and hydrogen content was reported for the first time. Furthermore and importantly, the results discuss the importance of hydrogenation in a‐Si QDs; future work will need to address the non‐trivial differentiation between surface H‐terminations and incorporation in the core (i.e., a‐Si:H QDs). The results evidence that hydrogenation plays an important role in parallel with quantum confinement and that the optical properties of a‐Si/a‐Si:H QDs can be tuned and enhanced by varying both size and hydrogen content. This has the potential of major impact on a range of applications where variable optical properties are required with abundant, nontoxic, and environmentally friendly materials.

## Supporting information

As a service to our authors and readers, this journal provides supporting information supplied by the authors. Such materials are peer reviewed and may be re‐organized for online delivery, but are not copy‐edited or typeset. Technical support issues arising from supporting information (other than missing files) should be addressed to the authors.

Supplementary

## References

[1] F. Priolo , T. Gregorkiewicz , M. Galli , T. F. Krauss , Nat. Nanotechnol. 2014, 9, 19. 24390564 10.1038/nnano.2013.271

[2] B. Ghosh , Y. Masuda , Y. Wakayama , Y. Imanaka , J. Inoue , K. Hashi , K. Deguchi , H. Yamada , Y. Sakka , S. Ohki , T. Shimizu , N. Shirahata , Adv. Funct. Mater. 2014, 24, 7151.

[3] P. Yu , C. Y. Tsai , J. K. Chang , C. C. Lai , P. H. Chen , Y. C. Lai , P. T. Tsai , M. C. Li , H. T. Pan , Y. Y. Huang , C. I. Wu , Y. L. Chueh , S. W. Chen , C. H. Du , S. F. Horng , H. F. Meng , ACS Nano 2013, 7, 10780. 24224917 10.1021/nn403982b

[4] N. Shehada , G. Bronstrup , K. Funka , S. Christiansen , M. Leja , H. Haick , Nano Lett. 2015, 15, 1288. 25494909 10.1021/nl504482t

[5] L. Tao , E. Cinquanta , D. Chiappe , C. Grazianetti , M. Fanciulli , M. Dubey , A. Molle , D. Akinwande , Nat. Mater. 2015, 10, 227. 10.1038/nnano.2014.325 25643256

[6] D. H. Shin , S. Kim , J. M. Kim , C. W. Jang , J. H. Kim , K. W. Lee , J. Kim , S. D. Oh , D. H. Lee , S. S. Kang , C. O. Kim , S.‐H. Choi , K. J. Kim , Adv. Mater. 2015, 27, 2614. 25776865 10.1002/adma.201500040

[7] D. Mariotti , S. Mitra , V. Švrcˇek , Nanoscale 2013, 5, 1385. 23334154 10.1039/c2nr33170e

[8] S. W. Kim , J. Lee , J. H. Sung , D. J. Seo , I. Kim , M. H. Jo , B. W. Kwon , W. K. Choi , H. J. Choi , ACS Nano 2014, 8, 6556. 24857043 10.1021/nn501683f

[9] M. L. Mastronardi , E. J. Henderson , D. P. Puzzo , G. A. Ozin , Adv. Mater. 2012, 24, 5890. 23289121 10.1002/adma.201202846

[10] K. Y. Cheng , R. Anthony , U. R. Kortshagen , R. J. Holmes , Nano Lett. 2011, 11, 1952. 21462935 10.1021/nl2001692

[11] H. A. Santos , E. Mäkilä , A. J. Airaksinen , L. M. Bimbo , J. Hirvonen , Nanomedicine 2014, 9, 535. 24787441 10.2217/nnm.13.223

[12] D. Timmerman , J. Valenta , K. Dohnalová , W. D. M. de Boer , T. Gregorkiewicz , Nat. Nanotechnol. 2011, 6, 710. 21984044 10.1038/nnano.2011.167

[13] M. C. Beard , K. P. Knutsen , P. Yu , J. M. Luther , Q. Song , W. K. Metzger , R. J. Ellingson , A. J. Nozik , Nano Lett. 2007, 7, 2506. 17645368 10.1021/nl071486l

[14] O. Wolf , M. Dasog , Z. Yang , I. Balberg , J. G. C. Veinot , O. Millo , Nano Lett. 2013, 13, 2516. 23662693 10.1021/nl400570p

[15] M. Dasog , G. B. De los Reyes , L. V. Titova , F. A. Hegmann , J. G. C. Veinot , ACS Nano 2014, 8, 9636. 25183018 10.1021/nn504109a

[16] K. Kusova , O. Cibulka , K. Dohnalova , I. Pelant , J. Valenta , A. Fucikova , K. Zidek , J. Lang , J. Englich , P. Matejka , P. Stepanek , S. Bakardjieva , ACS Nano 2010, 4, 4495. 20690596 10.1021/nn1005182

[17] V. Svrcek , K. Dohnalova , D. Mariotti , M. T. Trinh , R. Limpens , S. Mitra , T. Gregorkiewicz , K. Matsubara , M. Kondo , Adv. Funct. Mater. 2013, 23, 6051.

[18] D. M. Sagar , J. M. Atkin , P. K. B. Palomaki , N. R. Neale , J. L. Blackburn , J. C. Johnson , A. J. Nozik , M. B. Raschke , M. C. Beard , Nano Lett. 2015, 15, 1511. 25626139 10.1021/nl503671n

[19] S. Askari , I. Levchenko , K. Ostrikov , K. P. Maguire , D. Mariotti , Appl. Phys. Lett. 2014, 104, 163103.

[20] R. A. Street , Hydrogenated Amorphous Silicon, Cambridge University Press, New York, 1991.

[21] R. A. Street , Adv. Phys. 1981, 30, 593.

[22] Optoelectronics‐Materials and Techniques, (Ed: Predeep P.), InTech, Rijeka, Croatia, 2011; DOI: 10.5772/779.

[23] P. Rava , F. Demicheiis , G. Kaniadakis , P. Mpawenayo , A. Tagliaferro , E. Tresso , J. Vac. Sci. Technol. A 1987, 5, 1795.

[24] R. N. Kre , M. L. Mousse , Y. Tchetche , F. X. D. B. Bella , B. Aka , P. A. Thomas , Int. J. Phys. Sci. 2010, 5, 675.

[25] S. W. Hwang , C. H. Lee , H. Cheng , J. W. Jeong , S. K. Kang , J. H. Kim , J. Shin , J. Yang , Z. Liu , G. A. Ameer , Y. Huang , J. A. Rogers , Nano Lett. 2015, 15, 2801. 25706246 10.1021/nl503997m

[26] J. H. Park , L. Gu , G. V. Maltzahn , E. Ruoslahti , S. N. Bhatia , M. J. Sailor , Nat. Mater. 2009, 8, 331. 19234444 10.1038/nmat2398 PMC3058936

[27] N. M. Park , C. J. Choi , T. Y. Seong , S. J. Park , Phys. Rev. Lett. 2001, 86, 1355. 11178082 10.1103/PhysRevLett.86.1355

[28] R. Anthony , U. Kortshagen , Phys. Rev. B 2009, 80, 115407.

[29] T. Lopez , L. Mangolini , Nanoscale 2014, 6, 1286. 24326353 10.1039/c3nr02526h

[30] B. N. Jariwala , N. J. Kramer , M. C. Petcu , D. C. Bobela , M. C. M. van de Sanden , P. Stradins , C. V. Ciobanu , S. Agarwal , J. Phys. Chem. C 2011, 115, 20375.

[31] J. Holm , J. T. Roberts , J. Phys. Chem. C 2009, 113, 15955.

[32] D. Mariotti , V. Švrcˇek , J. W. J. Hamilton , M. Schmidt , M. Kondo , Adv. Funct. Mater. 2012, 22, 954.

[33] L. Mangolini , E. Thimsen , U. Kortshagen , Nano Lett. 2005, 5, 655. 15826104 10.1021/nl050066y

[34] C. M. Hessel , D. Reid , M. G. Panthani , M. R. Rasch , B. W. Goodfellow , J. Wei , H. Fujii , V. Akhavan , B. A. Korgel , Chem. Mater. 2011, 24, 393.

[35] D. Kovalev , J. Diener , H. Heckler , G. Polisski , N. Kunzner , F. Koch , Phys. Rev. B 2000, 61, 4485.

[36] A. Hadjadj , F. Larbi , M. Gilliot , P. R. I. Cabarrocas , J. Chem. Phys. 2014, 141, 084708. 25173031 10.1063/1.4893558

[37] D. C. Marra , W. M. M. Kessels , M. C. M. van de Sanden , K. Kashefizadeh , E. S. Aydil , Surf. Sci. 2003, 530, 1.

[38] G. Lucovsky , R. J. Nemanich , J. C. Knights , Phys. Rev. B 1979, 19, 2064.

[39] T. W. Kim , C. H. Cho , B. H. Kim , S. J. Park , Appl. Phys. Lett. 2006, 88, 123102.

[40] E. G. Barbagiovanni , D. J. Lockwood , P. J. Simpson , L. V. Goncharova , J. Appl. Phys. 2012, 111, 034307.

[41] Handbook of Thin Films Materials, Vol. 5: Nanomaterials and Magnetic Thin Films, (Ed: Nalwa H. S.), Academic Press, 2002.

