# Supplementary material for: The Interplay of Quantum Confinement and Hydrogenation in Amorphous Silicon Quantum Dots
Source: Adv Mater. 2015 Nov 2;27(48):8011–6. doi: 10.1002/adma.201503013 (PMC4738462; doi:10.1002/adma.201503013)
Supplement: Supplementary file 1 — Supplementary [file ADMA-27-8011-s001.pdf]

Copyright WILEY-VCH Verlag GmbH & Co. KGaA, 69469 Weinheim, Germany, 2015.

ADVANCED MATERIALS

Supporting Information

for *Adv. Mater.,* DOI: 10.1002/adma.201503013

The Interplay of Quantum Confinement and Hydrogenation in Amorphous Silicon Quantum Dots

Sadegh Askari, Vladmir Svrcek, Paul Maguire, and Davide Mariotti**

Supporting Information for

The Interplay of Quantum Confinement and Hydrogenation in Amorphous Silicon Quantum Dot

Sadegh Askari,[1] Vladmir Svrcek,[2] Paul Maguire,[1] Davide Mariotti[1]

[1]Nanotechnology & Integrated Bio-Engineering Centre-NIBEC, University of Ulster, UK

[2]Research Center for Photovoltaic Technologies, National Institute of Advanced Industrial Science and Technology-AIST, Japan

S1: Additional details on the plasma properties

For the synthesis of amorphous quantum dots (QDs), it is crucial to maintain the QDs temperature, during growth, below their crystallization temperature. Heating of QDs in atmospheric pressure plasma is due to interaction with the charged species (e.g. ions and electrons) while cooling is mainly due to the conduction to the surrounding gas.[19] The temperature of the gas in the plasma is generally always lower than the QDs temperature. Details of the gas temperature measurements can be found elsewhere.[19, S1-S2]

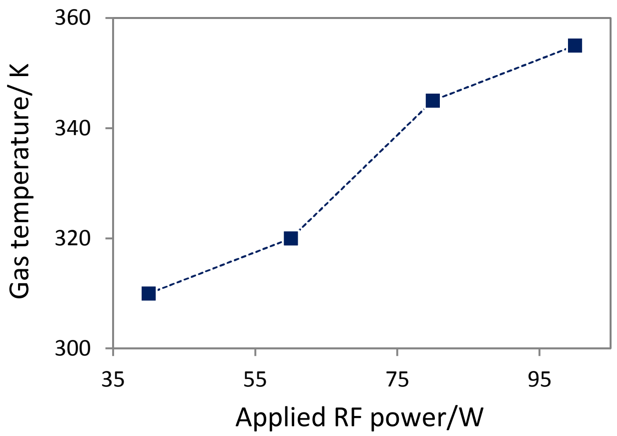

Figure S1. *Measured gas temperature as a function of the applied RF power.*

Figure S1 shows the measured gas temperature for argon plasma with flow rate 250 sccm versus applied RF power to the plasma; measurements were taken using the same plasma reactor described in the main manuscript. The result shows that the gas temperature is close to the room temperature and increases to the maximum value 355 K for the highest applied power. This value for gas temperature is well below the crystallization temperature reported for Si nanoparticles.[S3]

We should note that nucleation and growth in this plasma system is believed to proceed via atomization of the silicon atoms from silane.[19,S10] If the nanoparticle temperature is high, this allows for crystallization and unfavourable condition for the formation of Si-H bonds. However, if the temperature is low, hydrogen is not desorbed easily and therefore can be incorporated in the growing QDs, which do not have sufficient energy and suitable bonding arrangement to crystallize.

S2: Characterization of amorphous silicon quantum dots, no hydrogen added

Transmission electron microscopy (TEM) was employed for the analysis of the size, size distribution and crystal/amorphous structure of the a-Si QDs. Figure S2 shows the typical TEM images for samples with silane concentrations 10 ppm, 50 ppm (figure 2a-b in the main manuscript), 100 ppm and 200 ppm. The TEM analysis of the samples did not show any crystalline structure in any of the samples. The diffraction patterns that are shown in the insets of figure S2 also confirm the amorphous structure of the QDs.

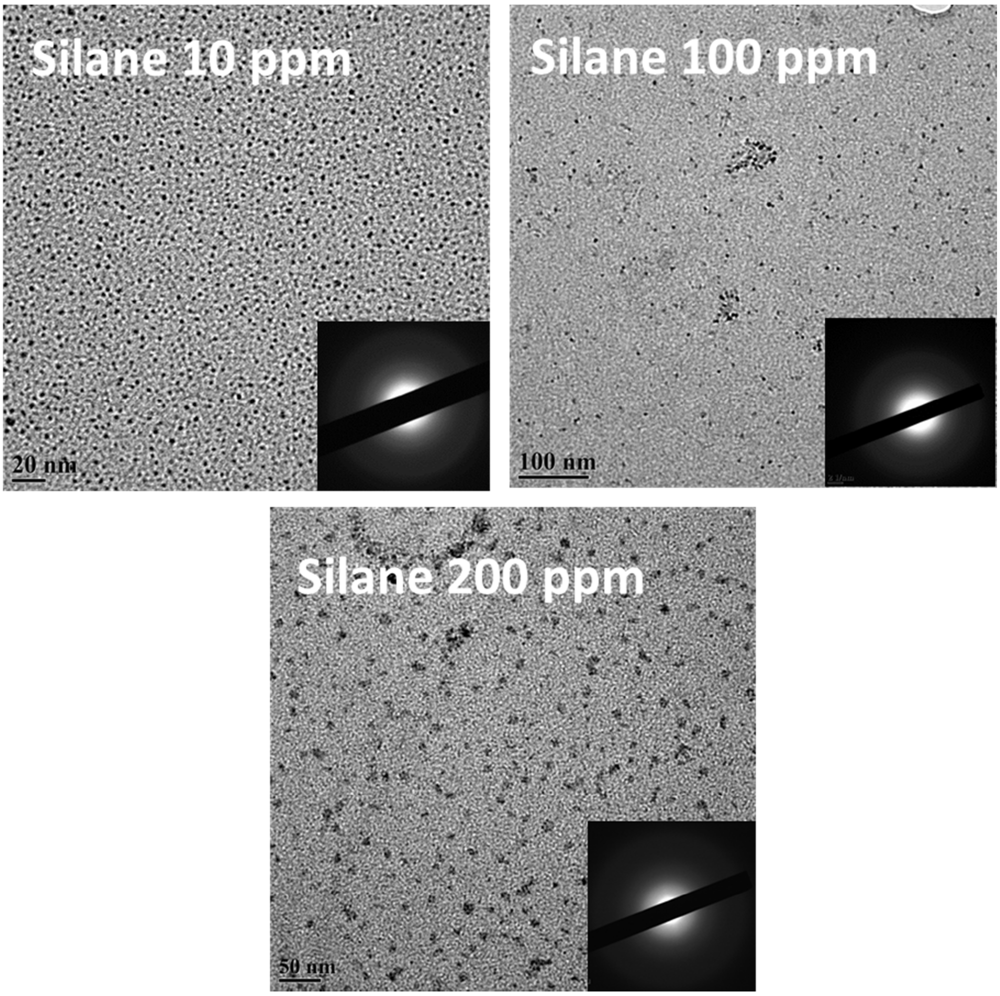

Figure S2. *Transmission electron microscopy images of amorphous Si quantum dots prepared at three different concentrations of silane 10 ppm, 100 ppm and 200 ppm. The corresponding diffraction patterns are displayed in the corresponding insets.*

TEM analysis includes two different and complementary types of measurements that support each other, i.e. imaging and diffraction patterns, the first based on the analysis of individual QDs and the second as a result of the electron beam interaction with an ensemble of QDs. These results from TEM analysis can therefore be considered conclusive on the amorphous nature of the Si QDs.

However, we have also performed Raman spectroscopy measurements (LabRam 300 Micro Raman spectrometer with a 632.8 nm helium-neon laser) and report the results for one of our samples. Figure S3 shows the Raman spectrum of the sample prepared with silane concentration at 10 ppm which produced Si QDs with mean diameter of 1.55 nm. The sample is prepared by pressing a thick layer of QDs powder on a piece of copper sheet. The spectrum is then collected using 1% of the laser power to avoid heating of the sample. The broad band observed around 478 cm^{-1} is associated with the transverse optical mode of a-Si [S9]. Also, no sharp peaks associated to crystalline silicon (close to 521 cm^{-1}) can be observed [S9].

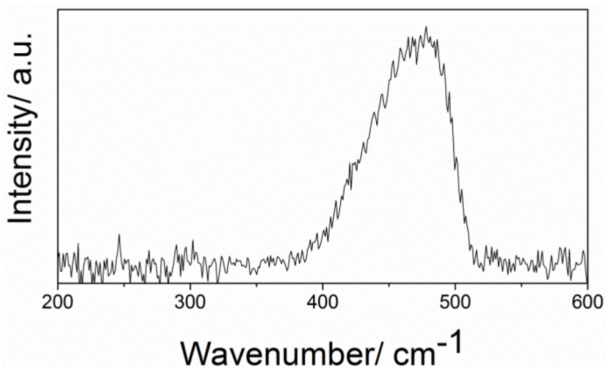

Figure S3. *Raman spectrum of the amorphous Si quantum with mean diameter of 1.55 nm prepared with a silane concentration of 10 ppm.*

The size distribution resulting from TEM analysis of the a-Si QDs is reported in figure S4 for the four different silane concentrations. These have shown mean diameters of 1.55 nm (10 ppm), 2.60 nm (50 ppm), 3.73 nm (100 ppm) and 5.15 nm (200 ppm).

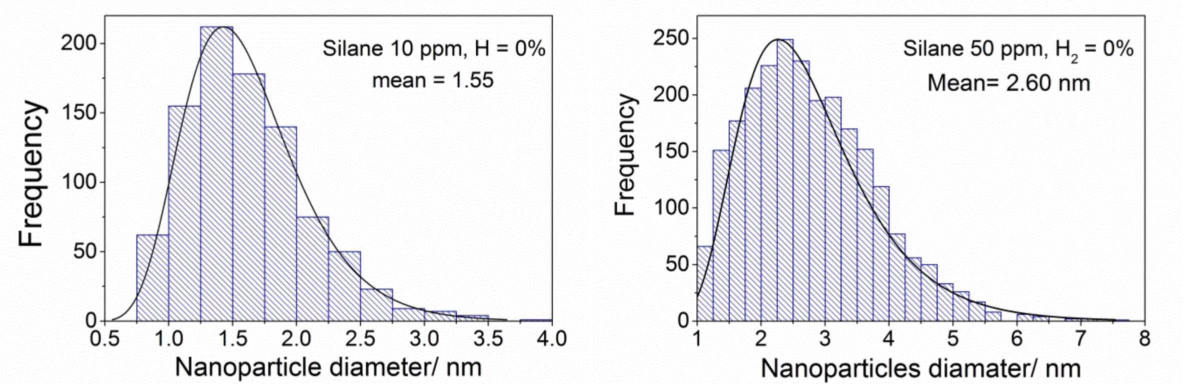

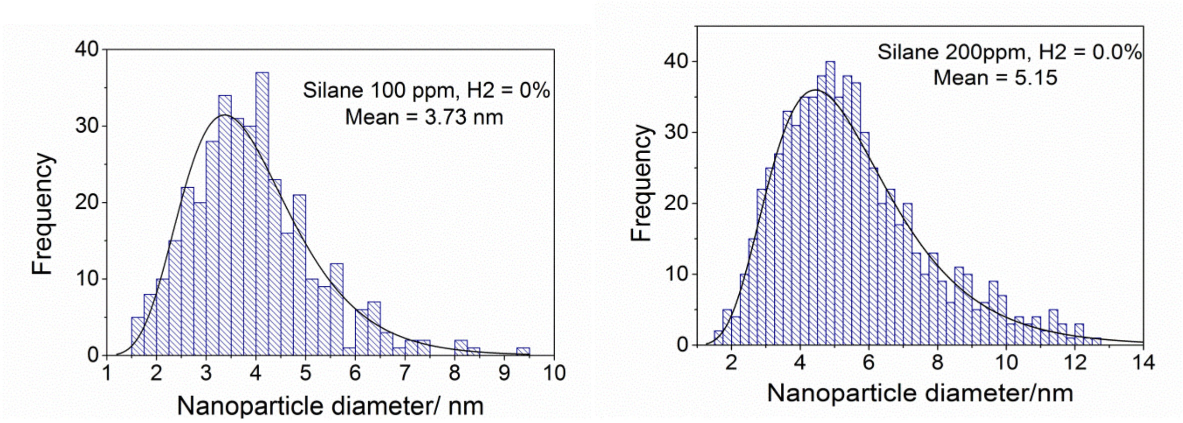

Figure S4. *Size distribution of the amorphous silicon quantum dots for different silane concentrations.*

The chemical composition of the a-Si QDs was evaluated with Fourier transform infrared spectroscopy (FTIR) (figure S5). Figure S5 includes FTIR from the QDs produced with the lowest (50 ppm) and highest (200 ppm) silane concentrations which exhibit a range of Si-H bonding arrangements confirming the QDs to be made of silicon with a degree of hydrogen content.

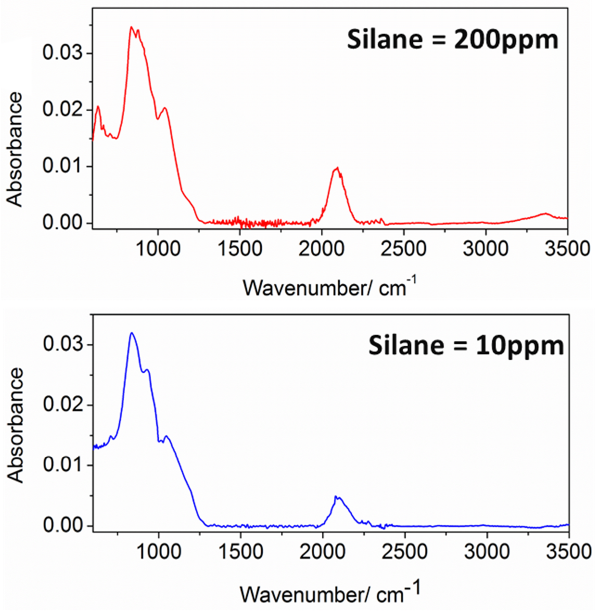

Figure S5. *Fourier transform infrared spectra for amorphous silicon quantum dots produced with the lowest and highest silane concentration used within this study.*

The spectra contain three characteristic band of silicon hydrides at 2000-2150 cm^{-1}, 830-920 cm^{-1} and at 630 cm^{-1}.[33-34] The doublet band at 830-920 cm^{-1} originates from different modes of higher hydrides (deformation modes of SiH_3, bend-scissor and wagging modes of $(SiH_2)_n$ etc. [33-34]). The dominance of this band in all the collected spectra represents abundance of higher hydrides rather than the monohydride (Si-H). The peak around 2100 cm^{-1} is from stretching modes of silicon hydride

including monohydride. The shoulder of the band at 1000-1100 cm^{-1} might originate from the silicon oxide as the sample has been exposed to the air (< 1 h) before characterization by FTIR. The low intensity of the silicon oxide peak at ~1100 cm^{-1} (and SiO-H at ~3700 cm^{-1}) confirms the very low degree of surface oxidation of the samples, as the intensity of these peak is commonly much stronger for Si QDs reported in the literature.[S4-S5] The FTIR results confirm the chemical composition of the a-Si:H QDs, with almost negligible surface oxidation and surface hydrogen terminations.

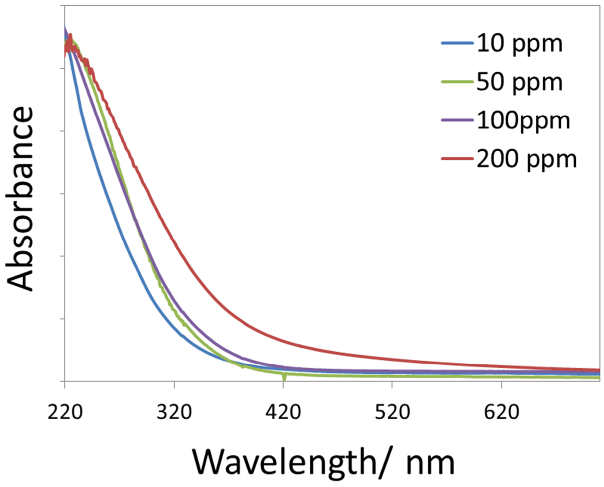

Figure S6. *Absorption spectra of the samples with four different concentrations of silane.*

Figure S6 displays the absorbance profiles of the colloids of a-Si QDs prepared at four different concentrations of silane. The absorption spectra can be used to determine the optical bandgap based on Tauc plots. Tauc plots can be produced for either direct or indirect bandgap behaviour. Based on the literature, an indirect bandgap behaviour has been assumed for a-Si QDs (figure S7).

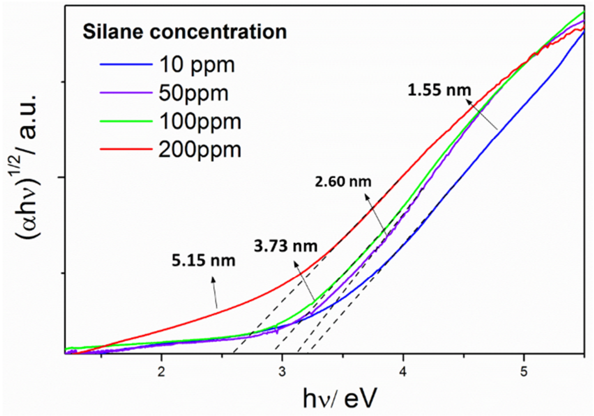

Figure S7. *Absorption spectra of the amorphous Si quantum dots at four different concentrations of silane in the range 10 ppm to 200 ppm plotted as* $(\alpha h\nu)^{1/2}$ *versus photon energy hν. The average size of the quantum dots is increased from 1.55 nm to 5.15 nm with increasing silane concentration. The absorption gap is estimated by extrapolation of straight portion of the profiles.*

However in figure S8 we report also the Tauc plot assuming a direct bandgap to show that also in this case the quantum confinement effect is evident. Bandgap values of 3.77eV, 3.58 eV, 3.48 eV and 3.18 eV for QDs with mean average 1.55 nm, 2.60 nm, 3.73 nm and 5.15 nm, respectively, were obtained.

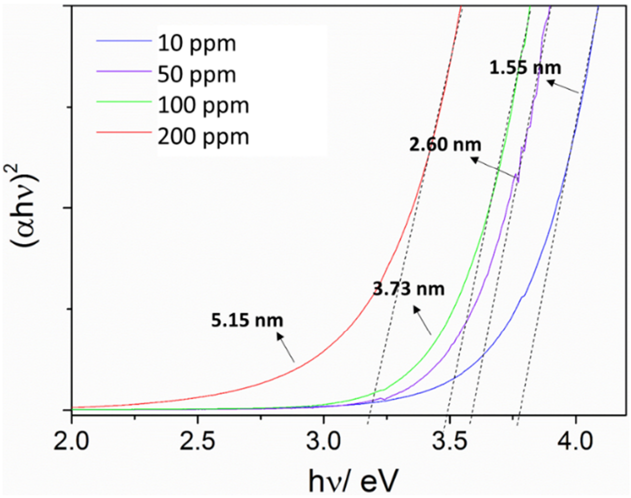

Figure S8. *Values of* $(\alpha h\nu)^2$ *versus photon energy hν calculated from absorption profiles for four different concentrations of silane in the range 10 ppm to 200 ppm. The direct absorption gap is estimated by extrapolation of straight portion of the profiles. In the plot α is the measured absorbance, h is Planck's constant and ν is the frequency.*

S3: Characterization of amorphous silicon quantum dots, with added hydrogen

TEM was employed for the analysis of the size, size distribution and crystal/amorphous structure of the a-Si QDs with added hydrogen. Figure S9 shows the typical TEM images for samples with 1.0% and 0.3% hydrogen concentration and 200 ppm silane (QDs with 0% hydrogen and 200 ppm silane was already reported at the bottom of figure S2). The TEM analysis of the samples did not show any crystalline structure in any of the samples. The diffraction patterns that are shown in the insets of figure S9 also confirm the amorphous structure of the QDs.

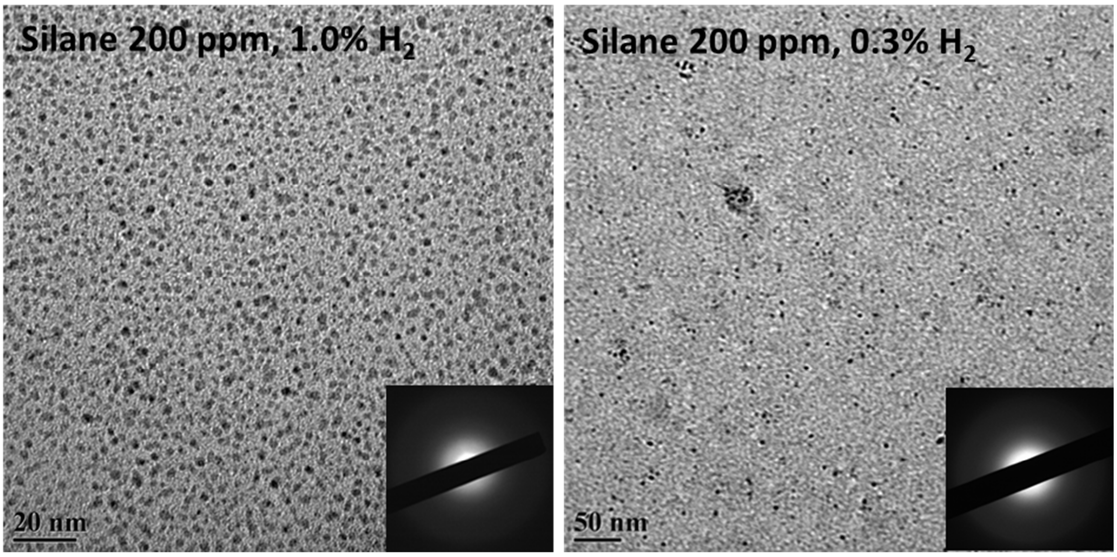

Figure S9. *Transmission electron microscopy analysis of amorphous silicon quantum dots with added hydrogen content.*

Also in this case we performed Raman spectroscopy to corroborate our TEM and diffraction pattern analysis. Figure S10 shows the Raman spectrum of the sample prepared with silane concentration at 200 ppm and 1% added hydrogen which produced Si QDs with mean diameter of 2.18 nm. The same procedure as above was followed for sample preparation and analysis. The broad band at 478 cm^{-1} is again measured with no sharp peaks associated to crystalline silicon above 500 cm^{-1}.

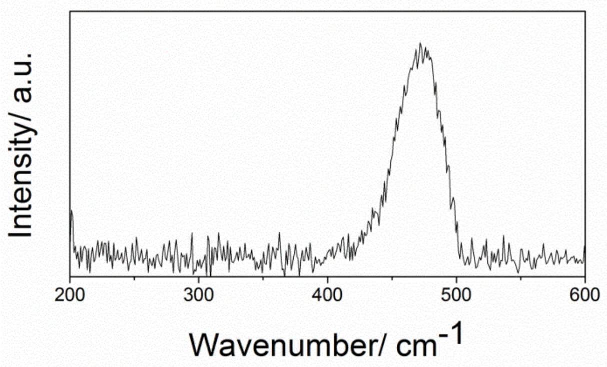

Figure S10. *Raman spectrum of the amorphous Si quantum with mean diameter of 2.18 nm prepared with a silane concentration of 200 ppm and 1% hydrogen gas.*

The size distribution from TEM analysis of the a-Si QDs with added hydrogen is reported in figure S11 for the two different hydrogen concentrations (QDs with 0% hydrogen and 200 ppm silane was already reported in figure S3). These have shown mean diameters of 5.15 nm (no hydrogen), 3.49 nm for 0.3% added hydrogen and 2.18 nm for 1.0% added hydrogen.

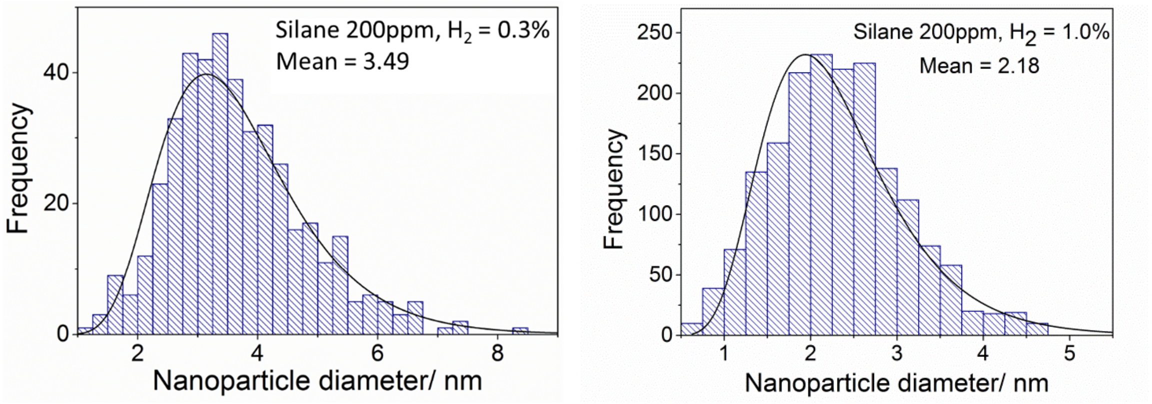

Figure S11. *Size distribution of amorphous quantum dots with added hydrogen prepared with 200 ppm silane and 1% (left) and 0.3% (right)* H_2.

FTIR analysis has provided also in this case the chemical composition of the amorphous QDs confirming the presence of various Si-H bonding arrangements analogous to figure S5. The FTIR spectra of the samples with three different concentrations of H_2 are displayed in figure S12. The silane concentration is 200 ppm for these samples.

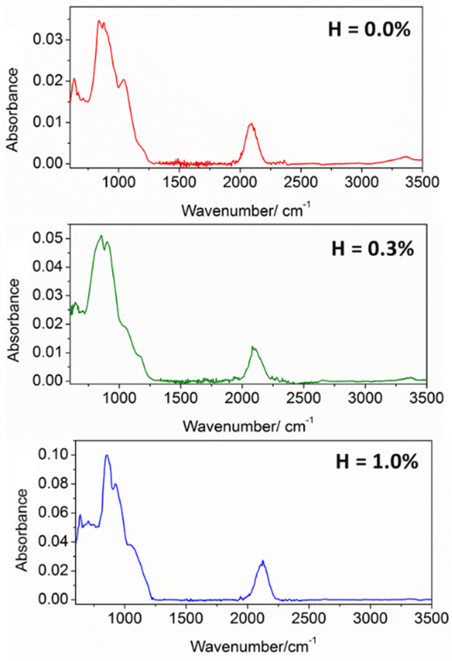

Figure S12. *Attenuated total reflectance Fourier transforms infra-red spectroscopy spectra of the samples at silane concentration 200 ppm and three different concentrations of* H_2.

Also in this case the FTIR results confirm the chemical composition of the a-Si QDs, with almost negligible surface oxidation and surface hydrogen terminations. However based on the discussion presented in the manuscript, an increased degree of hydrogenation is observed for these samples with diameters from 2.18 nm to 5.15 nm, which can be confirmed also by increased absorption intensities

of all peaks related to Si-H bonds as the hydrogen concentration is increased. In comparison, the FTIR signal of figure S5, which spans QDs with about the same mean diameters (1.55-5.15 nm) did not vary considerably suggesting the same level of hydrogenation.

S4: Estimation of the bandgap from absorption and photoluminescence measurements

The use of Tauc plots to estimate the bandgap have been widely used for bulk semiconductors. For nanoscale and in particular quantum confined systems, the evaluation of the bandgap using the same method presents some challenges and the introduction of assumptions and/or modified models.[S6] This is due in part to the possibility of transitions from indirect to direct (or vice versa) behaviour as well as to the low density of states close to the band-edges. While the qualitative trend of the bandgap values produced from Tauc plots is still valid, their absolute values might present non-negligible inaccuracies.

On this basis, photoluminescence measurements in some cases can be used to have more accurate estimates of the QDs bandgap. This is particularly true if surface characteristics have limited effect on the transition dynamics compared to quantum confinement effects. Quantum confinement generally tends to take over surface effects when the electronegativity of the terminating element is lower than that of silicon [S7-S8]; for instance, this is the case for hydrogen terminations where hydrogen low electronegative character limits the effect of surface transition dynamics and core-induced quantum confinement tend to dominate. Oxygen-based terminations on the other side have strong influence on the optical transitions affecting the outcome of photoluminescence measurements, which often present large Stokes shifts.

The material characterization of our Si QDs has shown very limited oxidation and predominant hydrogen-terminated surfaces. Therefore the use of photoluminescence to evaluate the QDs bandgap (in figure 5 of the main manuscript) appears to be justified in this case and supported by relatively small Stokes shifts (0.55-0.77 eV; see also main manuscript).

S5: Effective mass approximation fitting for hydrogenated amorphous silicon quantum dots

The effective mass approximation (EMA) model provide a relatively simple formula that describes the bandgap (E) in terms of the QD diameter (d) as

$$E = E_0 + \frac{A}{d^{\delta}} \qquad \text{Eq. (1)}$$

where E_0 is the bandgap of the material in its bulk form, A is a confinement factor and δ a factor generally equal to 2. Fitting of the experimental data (figure 5 in the main manuscript) for a-Si QDs (without added hydrogen) is relatively straightforward where good fitting was produced for values E_0 = 1.97 eV and A = 2.14 (E_0 is here the bulk bandgap for amorphous silicon with a degree of

hydrogenation deriving from silane). The degree of hydrogenation is assumed here constant as it directly depends on the plasma conditions (e.g. dissociation efficiency) which are very mildly changed by the different silane concentrations. This fitting procedure produced the blue line in figure 5 of the main manuscript where the values for the bandgap E_0 and A are close to those reported by experimental measurements and theoretical calculations for amorphous and hydrogenated silicon.[22-24,38]

The fitting procedure for a-Si QDs produced with varying hydrogen content (red line in figure 5 of the main manuscript) is somewhat more complicated because the bandgap value for a-Si (E_0) is expected to vary depending on the level of hydrogenation; specifically a larger bandgap is expected with increasing hydrogenation.[22-24] Therefore, we have determined different values for the bulk bandgap as in table 1 to reflect the increasing bulk bandgap as the hydrogen content is increased.

Table 1. *Bulk bandgap value used for curve fitting of the effective mass approximation model for amorphous silicon quantum dots with added hydrogen.*

**Added H_2 Concentration**	**Resulting QD diameter**	***QD bandgap E***	***Bulk bandgap E_0***
0.0%	5.15 nm	2.05 eV	1.97 eV
0.3%	3.49 nm	2.26 eV	2.09 eV
1.0%	2.18 nm	2.83 eV	2.38 eV

The values of the bulk bandgap in table 1 were obtained rearranging equation (1) using the corresponding experimental QD bandgap values (A = 2.14, as previously determined from the 0% added hydrogen case, and δ = 2). It is interesting to highlight that the bulk bandgap (E_0) values in table 1 were found to be in an almost perfect linear relationship with the added hydrogen concentration (figure S13), supporting further the hydrogenation of the a-Si QDs.

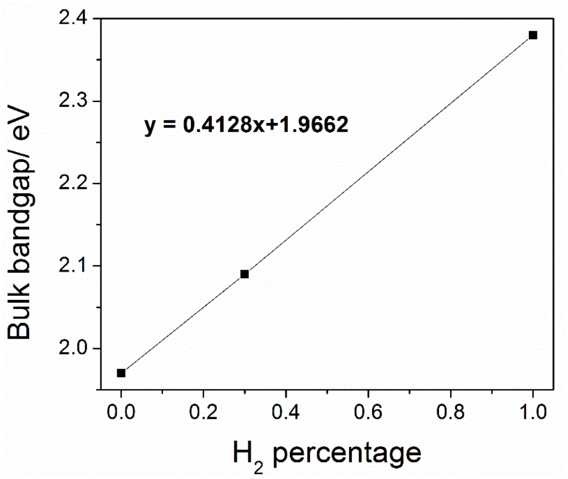

Figure S13. *Linear relationship between the added hydrogen concentration and the bulk bandgap.*

In order to produce the full theoretical EMA curve and interpolate points across the full range of sizes presented in figure 5 of the manuscript, the linear relationship from figure S13 ($E_0 = 0.41C_H + 1.97$, where C_H is the added hydrogen concentration) and the relationship between hydrogen concentration and QD size were used. The latter was determined empirically from experimental data in figure 2c of the main manuscript ($C_H = 0.12d^2 - 1.21d + 3.07$).

References

[S1] Hofmann, S.; Gessel, A. F. H. van; Verreycken, T.; Bruggeman, P. Plasma Sources Sci. Technol. **2011**, 20, 065010.
[S2] S. Y. Moon and W. Choe, Spectrochim. Acta, Part B **2003**, 58, 249.
[S3] Hirasawa, M.; Orii, T.; Seto, T. Appl. Phys. Lett. **2006**, 88, 093119.
[S4] Bywalez, R.; Karacuban, H.; Nienhaus, H.; Schulz, C.; Wiggers, H. Nanoscale Research Letters, **2012**, 7, 76.
[S5] Vladimirov, A.; Korovin, S.; Surkov, A.; Kelm, E.; Pustovoy, V. Laser Physics, 2011, 21, 830.
[S6] Feng, Y.; Lin, S.; Huang, S.; Shrestha, S.; Conibeer G. J. Appl. Phys. **2015**, 117, 125701.
[S7] Mariotti, D; Mitra, S.; Švrček, V. Nanoscale **2013**, 5, 1385.
[S8] Ramos, E.; Monroy, B. M.; Alonso, J. C.; Sansores, L. E.; Salcedo, R.; Mart´ınez, A. J. Phys. Chem. C, **2012**, 116, 3988.
[S9] Iqbal, Z; Veprek S. J. Phys. C: Solid State Phys. 1982, 15, 377.
[S10] Barwe, B.; Stein A.; Cibulka O. E.; Pelant I.; Ghanbaja J.; Belmonte T.; Benedikt J.; Plasma Processes Polym. 2015, 12, 132.
